# Supplementary material for: Polymer translocation through nano-pores in vibrating thin membranes
Source: Sci Rep. 2016 Dec 9;6:38558. doi: 10.1038/srep38558 (PMC5146916; doi:10.1038/srep38558)
Supplement: Supplementary Information [file srep38558-s1.pdf]

Supplementary Information for: Polymer translocation through nano-pores in vibrating thin membranes

Timothée Menais[1,2,3], Stefano Mossa[1,2,3], and Arnaud Buhot[1,2,3*]

[1]Univ. Grenoble Alpes, INAC-SYMMES, F-38000 Grenoble, France
[2]CNRS, INAC-SYMMES, F-38000 Grenoble, France
[3]CEA, INAC-SYMMES, F-38000 Grenoble, France
[*]arnaud.buhot@cea.fr

ABSTRACT

This document includes Supplementary Information for: *"Polymer translocation through nano-pores in vibrating thin membranes"*, by T. Menais, S. Mossa, and A. Buhot

Probability distributions of translocation times

In Fig. 1 we show a few examples of the probability distribution functions of the translocation times, for the cases of the narrow pore carved in an immobile membrane, at the indicated values of the pulling force f. These probability distributions are not Gaussian, and have been analyzed in terms of a first-passage probability density function, as suggested in Ref.[1] for the case of an electrophoretic bias,

$$F_1(\tau) = \frac{L}{\sqrt{4\pi D\tau^3}} e^{-(L-vt)^2/4D\tau}. \tag{1}$$

Here, τ is the translocation time, L the length of the polymer chain, D the diffusion coefficient of the membrane sliding along the polymer, and v the mean translocation velocity. We have adjusted Eq. (1) to the data, and the results are shown by the solid lines in the same Figure.

Waiting times in the pore

In Fig. 2 we show the single bead waiting time inside the pore as a function of the translocation coordinate (bead index) for a structured polymer with $N = 32$. The calculation of the waiting time includes possible returns in the pore from both cis- and trans- sides. The sum of the mean waiting times defines the mean translocation time. Data in the Figure refer to 10^3 translocation instances, under the effect of a pulling force $f = 12.83$, through the large and the narrow pores carved in an immobile membrane and discussed in the main text. We also show data pertaining to the linear polymer, as a reference. Data are renormalized to the average waiting time for each case.

In the case of the linear polymer, the curve is smooth, pointing to a translocation process where beads slides through the pore continuously. The case of the large pore presents a slight regular modulation of the waiting times pertaining to even/odd bead. This is due to the significant friction generated by the interaction of the base-like units with the pore. Those beads, we recall, are grafted to the sugar-like beads which correspond to even bead index along the chain. The general pattern of the translocation, still stays close to that observed for a linear polymer in Ref.[2]. The regular modulation correlated to the polymer structure is finally clear in the case of the narrow pore.

Translocation as a series of intermittent events

In the Top panel of Fig. 3 we show the number of beads which have translocated to the trans-side as a function of the elapsed time (MD steps) during a translocation event with $T_m = 0$, $k = 150$ and $f = 12.83$. In the Bottom panel of the same Figure, we plot the corresponding time dependence of the average displacements of the pore beads. These data have been calculated by separately considering the modules of the components (e.g., $(\delta x^2)^{1/2}$) averaged over all the beads forming the pore. We recall that the pulling force is applied in the $\hat{y}$-direction. In this particular translocation instance, displacements in the $\hat{y}$-direction are negligible, while those in the directions parallel to the membrane undergo intermittent impulsive variations, mirroring the

step-like translocation pattern. This observation correlates the translocation dynamics of the base-like beads to the dynamical state of the pore, and convincingly demonstrates the potential for sequencing applications.

Statistics of membrane beads displacements during translocation

In Fig. 4 we plot the probability distributions for the displacements of a bead pertaining to the membrane and chosen at random, at the indicated values of T_m (expressed in terms of $T_p = 3/2$ in all cases), for $k = 150$ and $f = 12.83$. Here we have chosen to plot the data pertaining to the components of the displacements (e.g., δx, δy, δz). Dynamics is isotropic in this case, implying that we can extract a single histogram from the three components. These data have been calculated from 400 translocation instances. The dynamics of membrane beads further from the pore are not significantly impacted by interaction with the translocating polymer, and simply undergo small oscillations around the equilibrium position due to thermal fluctuations. For the central limit theorem they therefore conform to a Gaussian probability distribution. We show the result of Gaussian fits to the data by solid line. The corresponding standard deviations (σ) are shown in the *Bottom* panel, as a function of T_m/T_p. We also show (solid line) a fit of the form $\sigma \propto (T_m/T_p)^{1/2}$, as implied by the equipartition theorem.

In the Top panel of Fig. 5 we show the probability distributions for the components of the displacements of one random bead pertaining to the pore, averaged over 400 translocation instances at the indicated membrane temperatures T_m (expressed in terms of T_p) for $k = 150$ and $f = 12.83$. At variance with the case of Fig. 4, these distributions are non-Gaussian, and feature a non-zero mean, due to the non-equilibrium dynamical processes associated to the interaction with the polymer which add to thermal fluctuations. Still, the standard deviation seems again to conform to the $\sigma \propto (T_m/T_p)^{1/2}$ prescription of the equipartition theorem for $T_m > 0$ (see Bottom panel). In the same Figure we also show the point corresponding to $T_m = 0$, where the pore deformation only is present. A complete understanding of these data is clearly an interesting issue, which goes, however, beyond the scope of the present paper.

References

1. Ling, D. Y. & Ling, X. S. On the distribution of DNA translocation times in solid-state nanopores: an analysis using schrödinger's first-passage-time theory. *Journal of Physics: Condensed Matter* **25**, 375102 (2013). URL http://dx.doi.org/10.1088/0953-8984/25/37/375102.

2. Kantor, Y. & Kardar, M. Anomalous dynamics of forced translocation. *Physical Review E* **69**, 021806 (2004). URL http://dx.doi.org/10.1103/PhysRevE.69.021806.

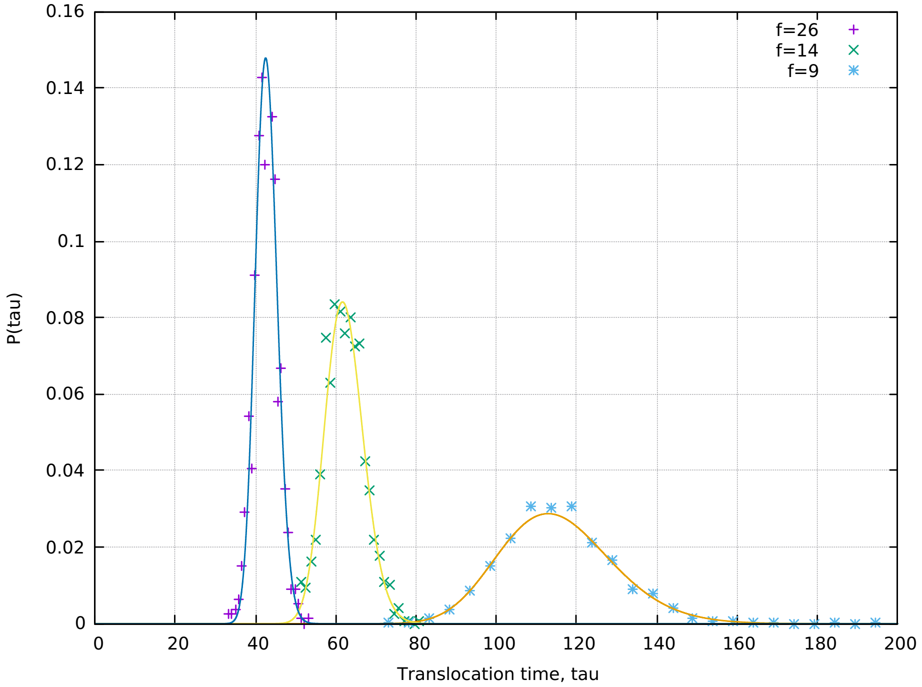

Figure 1. Translocation time distributions for the structured polymer moving through the immobile narrow pore, at the indicated values of f. We also show as solid lines the fits to the data according to Eq. (1).

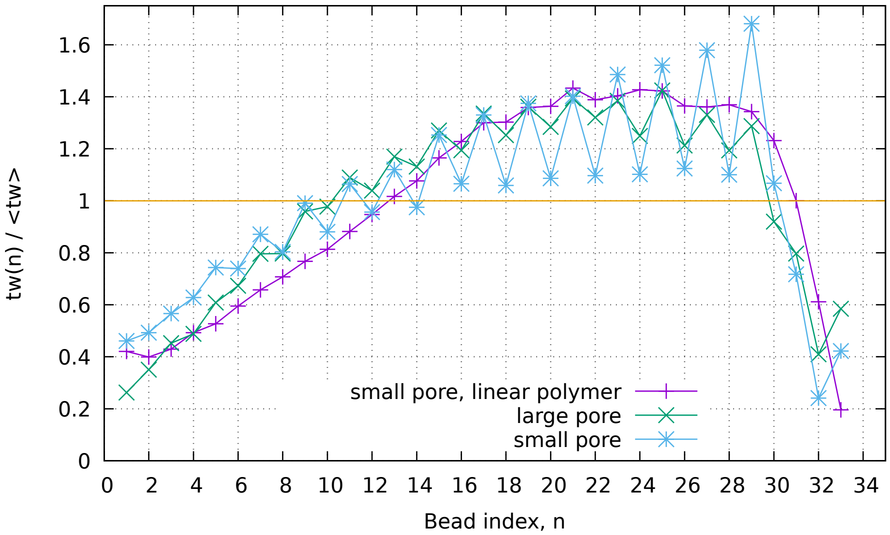

Figure 2. Single bead waiting time inside the pore as a function of the translocation coordinate (bead index) for a structured polymer with $N = 32$. Data refer to 10^3 translocation instances, under the effect of a pulling force $f = 12.83$, through a large and a narrow pore carved in an immobile membrane, as discussed in the main text. We also show results pertaining to the linear polymer, as a reference. Data are renormalized to the average waiting time for each case.

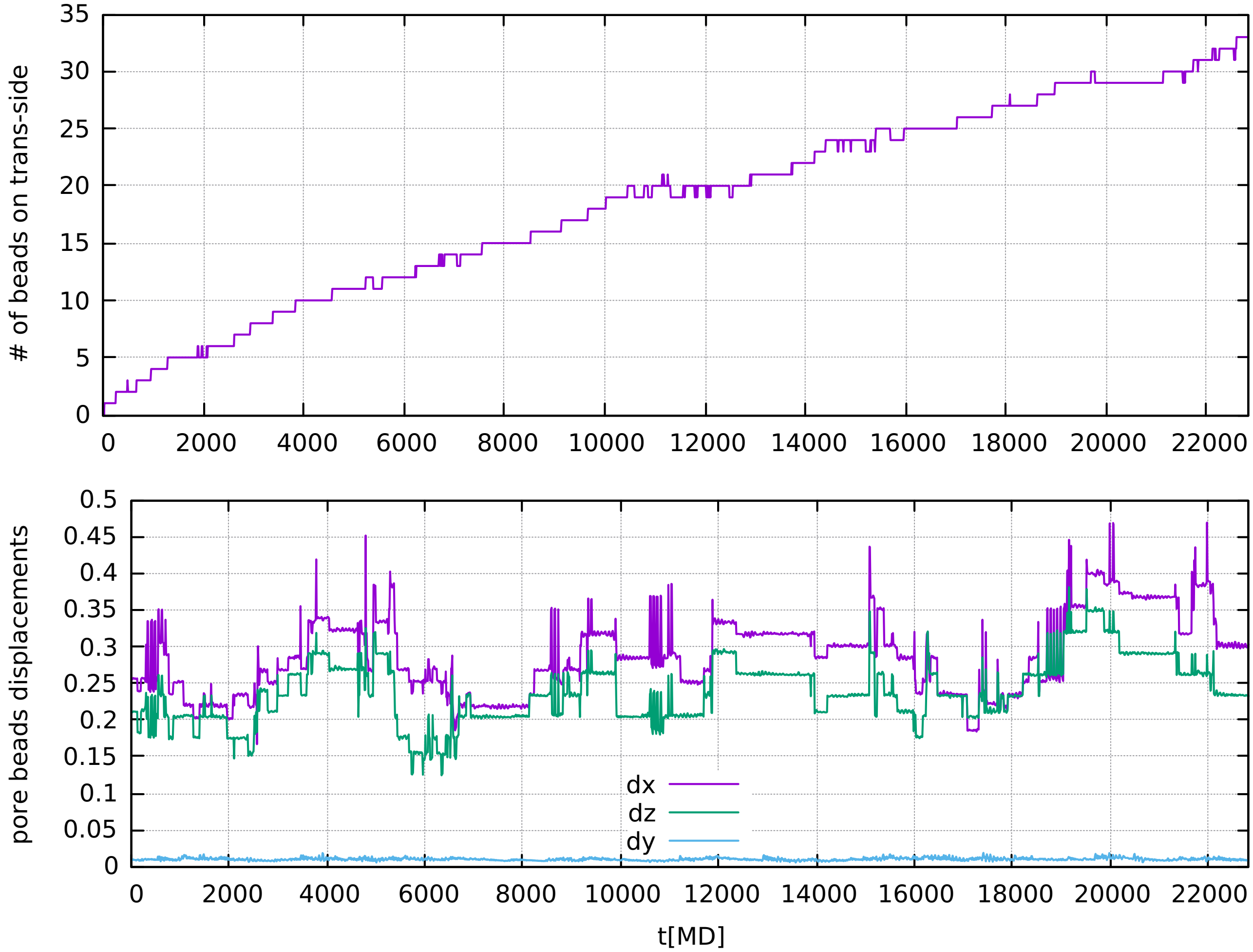

Figure 3. *Top:* Number of beads having translocated to the trans-side as a function of time (MD steps) during a single translocation event ($T_m = 0$, $k = 150$ and $f = 12.83$). *Bottom:* Corresponding time dependence of the average displacements of the beads forming the pore. These data have been calculated by separately considering the modules of the components (e.g., $(\delta x^2)^{1/2}$) averaged over all the beads forming the pore. We recall that the pulling force is applied in the $\hat{y}$-direction.

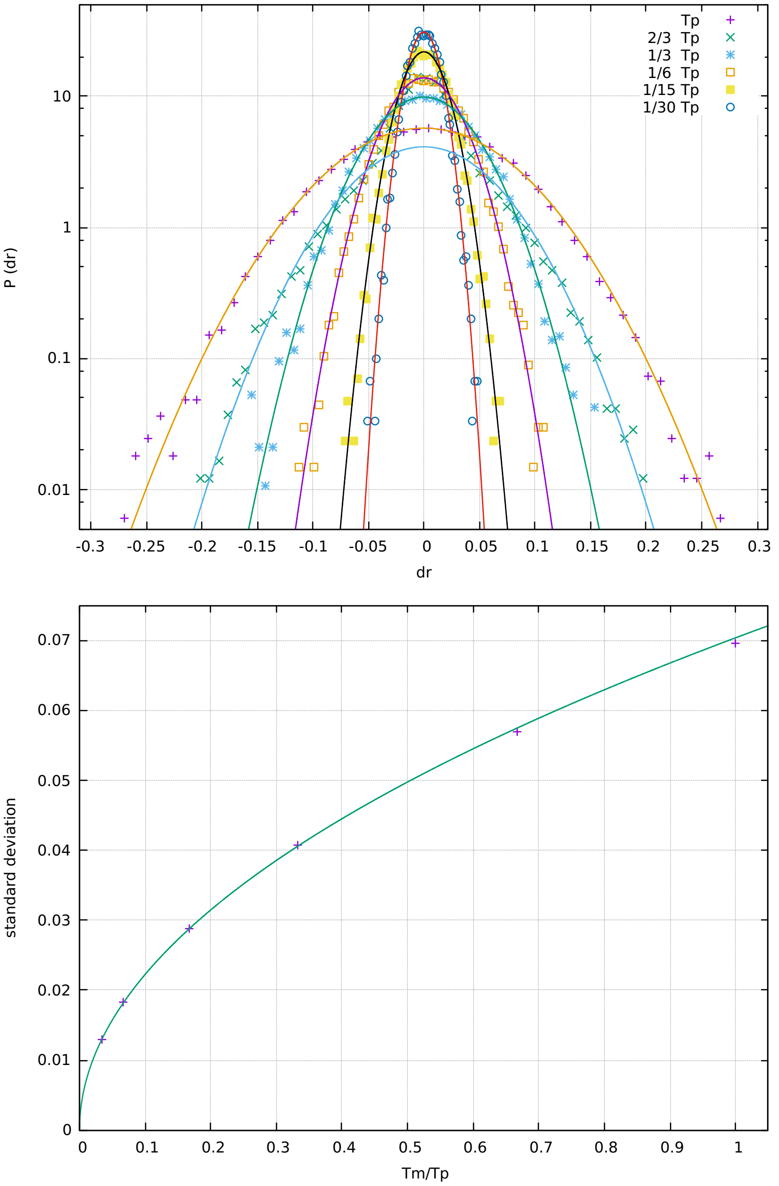

Figure 4. *Top:* Probability distributions for the displacements of a bead pertaining to the membrane and chosen at random, at the indicated values of T_m (expressed in terms of T_p), for $k = 150$ and $f = 12.83$. These data have been calculated from 400 translocation instances. We also plot the result of Gaussian fits to the data, shown by solid lines. The corresponding standard deviations (σ) are shown in the *Bottom* panel, as a function of T_m/T_p. We also show (solid line) a fit of the form $\sigma \propto (T_m/T_p)^{1/2}$, as prescribed by the equipartition theorem.

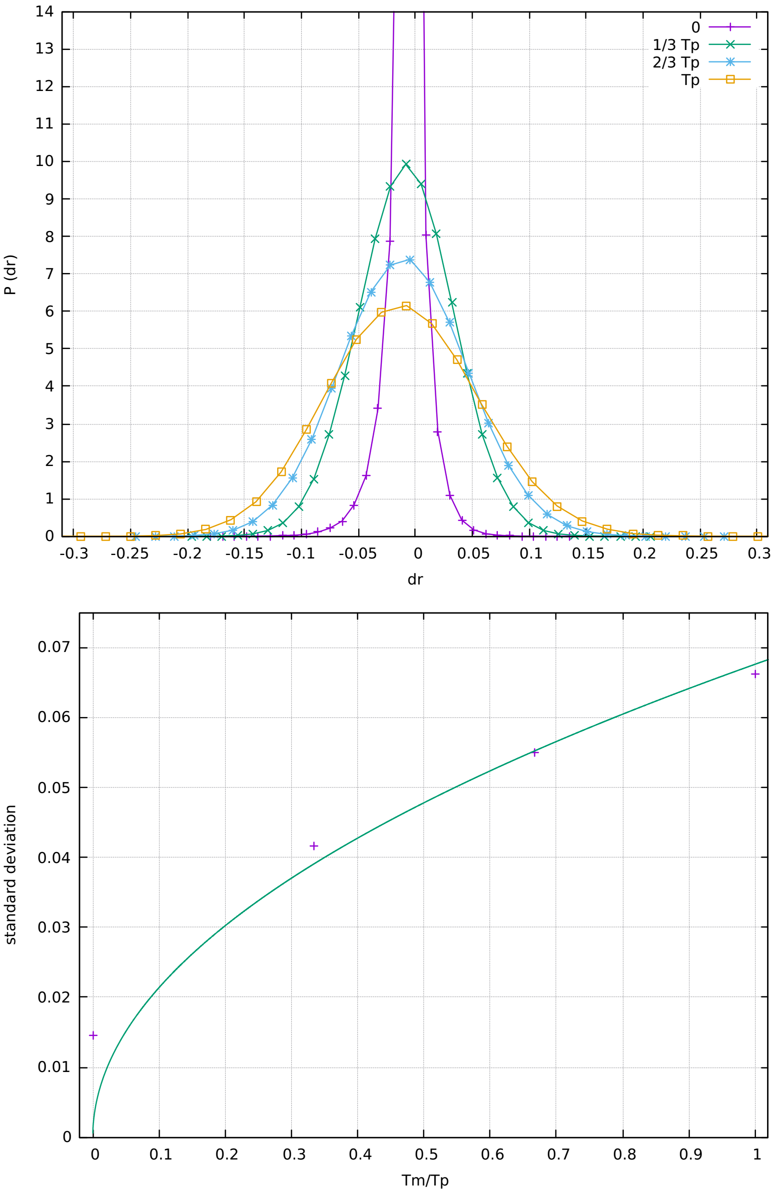

Figure 5. *Top:* Probability distributions for the displacements of a random bead pertaining to the pore, at the indicated values of T_m (expressed in terms of T_p), for $k = 150$ and $f = 12.83$. These data have been calculated from 400 translocation instances. At variance with the case of Fig. 4, these distributions are non-Gaussian, with a mean shifted to non-zero values, due to the non-equilibrium dynamical processes associated to the interaction with the polymer which add to thermal fluctuations. *Bottom:* The standard deviations of the distributions conform to the $\sigma \propto (T_m/T_p)^{1/2}$ prescription of the equipartition theorem. We also show the point corresponding to $T_m = 0$, where the pore deformation only is present.
